# MicroRNAs Related to Polycystic Ovary Syndrome (PCOS)

**DOI:** 10.3390/genes5030684

**Published:** 2014-08-25

**Authors:** Anja Elaine Sørensen, Marie Louise Wissing, Sofia Salö, Anne Lis Mikkelsen Englund, Louise Torp Dalgaard

**Affiliations:** 1 Department of Science, Systems and Models, Roskilde University, Universitetsvej 1, Roskilde 4000, Denmark; E-Mails: sofias@ruc.dk (S.S.); ltd@ruc.dk (L.T.D.); 2 Fertility Clinic Region Sjaelland, Holbaek Hospital, Holbaek 4300, Denmark; E-Mails: mlwi@regionsjaelland.dk (M.L.W.); aleg@regionsjaelland.dk (A.L.M.E.)

**Keywords:** polycystic ovary syndrome, microRNA, biomarkers, insulin resistance, infertility, diabetes

## Abstract

Polycystic ovary syndrome (PCOS) is the most common, though heterogeneous, endocrine aberration in women of reproductive age, with high prevalence and socioeconomic costs. The syndrome is characterized by polycystic ovaries, chronic anovulation and hyperandrogenism, as well as being associated with infertility, insulin resistance, chronic low-grade inflammation and an increased life time risk of type 2 diabetes. MicroRNAs (miRNAs) are small, non-coding RNAs that are able to regulate gene expression at the post-transcriptional level. Altered miRNA levels have been associated with diabetes, insulin resistance, inflammation and various cancers. Studies have shown that circulating miRNAs are present in whole blood, serum, plasma and the follicular fluid of PCOS patients and that they might serve as potential biomarkers and a new approach for the diagnosis of PCOS. In this review, recent work on miRNAs with respect to PCOS will be summarized. Our understanding of miRNAs, particularly in relation to PCOS, is currently at a very early stage, and additional studies will yield important insight into the molecular mechanisms behind this complex and heterogenic syndrome.

## 1. Introduction

Polycystic ovary syndrome (PCOS) is a complex, multifactorial endocrine disorder affecting approximately 5% to 10% of all women of reproductive age [1,2]. PCOS can be defined based on three different criteria; the National Institutes of Health (NIH) [3], the European Society of Human Reproduction and Embryology/American Society for Reproductive Medicine (ESHRE/ASRM, Rotterdam) [4] and the Androgen Excess Society (AES) [5]. Depending on which criteria are used, as well as the studied population, the prevalence of PCOS in women at reproductive age differs from 6% to 8%, using the NIH criteria, and up to 20%, using the Rotterdam criteria [2,6,7]. All of the criteria include, to some extent, anovulation, hyperandrogenism (clinical and/or biochemical) and polycystic ovaries with the exclusion of other androgenic, pituitary or adrenal causes. Aside from being associated with infertility and obesity, PCOS is also associated with an increased life time risk of developing type 2 diabetes (T2D), insulin resistance, hypertension, oxidative stress, dyslipidemia, as well as cardiovascular diseases [5]. The etiology of PCOS is still unclear, but environmental and genetic factors may contribute to the pathogenesis of PCOS. It may be explained by the existence of a vicious perpetual circle of pathological effects, where androgen excess favouring visceral abdominal fat disposition facilitates an increased secretion of androgens by the ovaries and/or the adrenal glands [8]. Insulin resistance, a common feature of PCOS, but not a diagnostic criterion of PCOS, leads to compensatory hyperinsulinemia with diverse effects on adipose tissue and androgen production. The prevalence of insulin resistance in women with PCOS is as high as up to seventy percent [5].

MicroRNAs (miRNA) are endogenous, small, non-coding, single-stranded, regulatory RNA molecules, composed of 20–24 nucleotides [9]. Processed from larger stem-loop precursor transcripts, miRNAs modulate gene expression post-transcriptionally by binding to the 3' untranslated region (UTR) of target mRNA, thus inhibiting translation, inducing mRNA destabilization, or both [10]. Recent studies have shown that miRNA can be encapsulated in microvesicles [11,12,13,14] or be free-circulating [15], and they have been isolated from serum [16], plasma [17], urine [19], saliva [11] and semen [19].

Little is known about the roles of miRNAs during follicular development, steroidogenesis and in PCOS. Several studies on miRNA expression have been done on intact ovaries (chicken [20], mice [21,22,23,24], pig [25,26], sheep [27] and cattle [28,29]), as well on the different ovarian components, such as granulosa cells (mice [30,31,32], pig [33], horses [14] and human [34,35]), theca cells [32], follicular fluid (humans [15,36], cattle [32] and mares [14,37]), cumulus cells (mare [14]), cumulus-oocyte complexes (COCs) (cattle [32,38]) and corpora lutea (cattle [39]).

The possible modes of action for miRNA within the pathophysiology of PCOS have only been sparsely investigated, and thus far, only a few miRNA-PCOS studies exist (see Table 1). The purpose of this review is to discuss the possible roles of miRNAs in polycystic ovary syndrome, insulin resistance and infertility. A greater understanding of the underlying molecular mechanism may help to improve diagnosis and treatment of this syndrome.

**Table 1:** List of microRNAs observed in polycystic ovary syndrome (PCOS), their proposed functions and targets.

microRNA	Detected in Tissue/Cell	Species	Target Gene (s)	Reported Function(s)	Observation in PCOS	References
**miR-9**	Follicular fluid Granulosa cells	Human	IL8, SYT1, IRS2	Inhibits testosterone release. Increases expression of PCNA	Significantly increased expression in PCOS	[34,35,36]
**miR-18b**	Follicular fluid Granulosa cells	Human	IL8, SYT1, IRS2	Promotes progesterone release while inhibiting testosterone and estradiol release; decreases PCNA expression; promotes Bax expression	Significantly increased expression in PCOS	[34,35,36]
**miR-19b**	Blastocysts	Human			Significantly decreased expression in PCOS blastocysts	[40]
**miR-21**	Whole blood Follicular fluid Granulosa cells	Human Mouse Rat		Reduced in obesity and type 2 diabetes; anti-apoptotic; increased expression after FSH exposure; inconclusive testosterone response	Increased expression in PCOS whole blood	[13,31,35,41,42]
**miR-27b**	Whole blood	Human		Hormone metabolism; inflammation; adipogenesis; reduced in obesity Positively correlated with testosterone	Increased expression in PCOS.	[41]
**miR-30c**	Serum Granulosa cells	Human Rat		Increased expression after FSH exposure	Significantly increased expression in PCOS	[42,43]
**miR-93**	Blastocysts Adipocytes	Human	SIRT1, GLUT4	Inhibits SIRT1 and GLUT4	Significantly decreased expression in PCOS blastocysts, while over-expressed in adipose tissue	[40,44]
**miR-103**	Whole blood Granulosa cells	Human		Promotes progesterone release while inhibiting estradiol release; hormone metabolism; reduced in obesity	Increased expression in PCOS; positively correlated with testosterone	[35,41]
**miR-132**	Follicular fluid Granulosa cells Granulosa-like tumor cell line(KGN)	Human Mouse Rat	HMGA2, Ctbp1	Increases estradiol secretion and reduces progesterone and testosterone release; increased expression during hCG-induced ovulation; increases PCNA expression; decreases Bax expression; increased expression after FSH exposure.	Significantly decreased expression in PCOS	[15,30,34,35]
**miR-135a**	Follicular fluid Granulosa cells	Human	IL8, SYT1, IRS2	Reduces progesterone and testosterone release; decreases Bax expression	Significantly increased expression in PCOS	[34,35,36]
**miR-146a**	Serum Plasma Follicular fluid Granulosa cells	Human		Reduces progesterone, estradiol and testosterone release	Significantly increased serum expression in PCOS; present in follicular fluid of PCOS women	[15,35,43]
**miR-155**	Serum Granulosa cells	Human		Inhibits testosterone release; decreases PCNA expression; decreases Bax expression	Increased serum expression in PCOS	[34,35,41]
**miR-222**	Serum follicular fluid Ovary Granulosa-like tumor cell line(KGN)	Human Rat	Estrogen receptor 1	Associated with type 2 diabetes; positively correlated with serum insulin; increases estradiol secretion	Significantly increased serum expression in PCOS; present in follicular fluid of PCOS women; present in rat TCs	[15,24,43,45]
**miR-224**	Follicular fluid Cumulus-oocyte complex Granulosa cells	Human Mouse	PTX3, Smad4	Inhibits PTX3; induces GCs proliferation through TGF-β1; increases estrogen release	Differentially expressed in follicular fluid	[36,46,47]
**miR-320**	Serum Follicular fluid Granulosa cells Adipocytes	Human Mouse	RAB5B, E2F1, SF-1	Down-regulated when treated with TGF-β1 Increased in insulin resistance	Decreased serum expression in PCOS and inconsistent expression in follicular fluid in PCOS; increased expression granulosa cells	[15,43,48,49]
**miR-383**	Follicular fluid Granulosa cells Oocyte	Human Mouse	RBMS1	Enhances the release of estradiol from GCs by CYP19A1; downregulation induced by TGF-β1	Higher expression found in PCOS women	[36,48,50]

## 2. MicroRNA Biogenesis and Function

The biogenesis, function and action of miRNAs has been extensively and well-covered elsewhere [51,52,53]. Briefly, a primary miRNA (pri-miRNA) is transcribed from the genome by RNA polymerase II or III, forming an imperfect stem-loop hairpin structure up to several hundred nucleotides (nt) in length. A nuclear protein complex consisting of the Drosha enzyme together with several other proteins is responsible for the cleavage of pri-miRNA, yielding a precursor miRNA (pre-miRNA) transcript of 60–110 nt in length that can be exported out of the nucleus by exportin-5 (XPO5) into the cytoplasm. Another protein complex containing the enzyme Dicer subsequently processes the pre-miRNA further, giving rise to a double-stranded miRNA complex containing the mature miRNA and a passenger strand. The mature miRNA associates with the miRNA-induced silencing complex (RISC) and targets the 3' untranslated region (3'UTR) of mRNA. Whether the target mRNA is degraded, or translation is repressed, depends on the complementarity between the miRNAs seed region (positioned from base 2 to base 8–9) and the sequence of the mRNA 3'UTR. Higher complementarity more often results in degradation rather than inhibited translation [54].

It is well established that protein-encoding genes can be modified through epigenetic mechanisms; however, epigenetic modifications have also been observed for a subset of miRNA-encoding genes [55,56,57].

Epigenetic modifications refer to stable heritable changes in gene activity and expression that are not caused by alterations in the DNA sequence itself. Genes’ availability for transcription and, thereby, their expression can be influenced by methylation, chromatin remodeling and acetylation. Furthermore, small RNAs, including miRNAs, can also act in an epigenetic manner, causing changes in gene expression [58].

It might be possible that identified PCOS susceptibility genes, such as *DENND1A* [59], which interestingly also encodes miR-601 [60], could result in genetic and epigenetic factors overlapping, thus influencing miRNA target specificity. A pilot study investigating global methylation in twenty PCOS women and 20 BMI- and age-matched controls, using peripheral leukocyte DNA, showed no significant differences in the median global DNA methylation percentages [61]. Despite the negative result, epigenetics may still play a part in PCOS pathogenesis, since GC and ovary gene-expression could be tissue specifically epigenetically modified in PCOS. Thus, PCOS is genetically complex with a large degree of heterogeneity and is considerably influenced by environmental and genetic cues, one of these being microRNAs.

## 3. Serum/Plasma miRNA Biomarkers for PCOS

Present abundantly in serum, miRNAs could serve as a non-invasive biomarker for PCOS, as they have been shown to be stable in serum, are resistant to nuclease activity and are easy to detect [62]. It is not known specifically how miRNAs enter serum or whether the miRNAs present in serum are disease-specific, since serum is a result of different components secreted by various tissues and cells, and identifying their cellular origin can be difficult. Currently, several other biomarkers in the serum of PCOS women are used for diagnostic purposes, e.g., luteinizing hormone (LH) and androgen concentrations, as well as follicle-stimulating hormone (FSH) [7,63].

A recent case-control study investigating 12 PCOS patients, 12 healthy females and 12 male controls, subdivided further based on BMI levels, revealed that obesity significantly reduced the expression of four miRNAs selected for evaluation in whole blood: miR-21, miR-27b, miR-103 and miR-155 in control women and men, but tending to show an increase in expression in PCOS women. Further analysis of their hormone profile showed a positive correlation between serum free testosterone levels and miR-21, miR-27b and miR-155. Perhaps, the elevated free testosterone found in the PCOS samples could partly explain the observed increase of these miRNAs. Further, bioinformatics analysis and target gene analysis revealed that miR-21, miR-27b, miR-103 and miR-155 could be involved in hormone metabolism, as well as reproductive cellular processes [41].

Using miRNA arrays, the expression of serum miRNAs in patients with PCOS compared to age-matched controls has been evaluated [43]. Following an initial miRNA profiling based on a relative two-fold change in expression levels, nine miRNAs (miR-222, miR-16, miR-19a, miR-106b, miR-30c, miR-146a, miR-24, miR-186 and miR-320) were chosen for further analysis. The expression levels for eight of the miRNAs were upregulated in serum from PCOS patients, whereas miR-320 displayed decreased expression in the PCOS subjects. However, following Q-PCR validation of the nine miRNAs’ expression in the entire study population (*n* = 68 PCOS, *n* = 68 controls), only miR-222, miR-146a and miR-30c remained significantly increased in the PCOS patients’. Sensitivity and specificity analysis, using receiver operating characteristic (ROC) curves and area under the curve (AUC), revealed that a combination of the three miRNAs was able to distinguish between the PCOS and controls. In addition, correlation analysis adjusted for age and BMI showed that miR-222 strongly correlated positively with serum insulin levels in PCOS women [43]. Interestingly, upregulated expression levels of miR-222 have also been associated with type 2 diabetes [64] and gestational diabetes mellitus [65]. Further, miR-146a correlated negatively with serum testosterone in PCOS women [43]. Decreased miR-146 has been linked to inflammation and insulin resistance in T2D individuals [66]. An interesting observation made by Long *et al.* was that most of the miRNAs present and differentially expressed in ovarian tissue from PCOS women were not released into the blood and, therefore, were not altered in PCOS serum [43].

In conclusion, identification of distinct miRNAs present within the circulation would prove a useful tool for diagnosis and perhaps treatment of PCOS. Comparing the miRNA profile of PCOS patients to healthy controls reveals that miRNAs might contribute to the pathogenesis. Indeed, miR-21, miR-27b and miR-103 are associated with PCOS, as well as metabolic features, such as obesity, T2D, low-grade inflammation and adipogenesis dysfunction. Furthermore, insulin sensitivity and the suppression of androgens have been associated with miR-222 and miR-146a, respectively. Profiling of serum miRNAs does not necessarily reflect the more local changes within the ovary, and the functional role and significance of miRNAs in blood from PCOS patients still need to be determined.

## 4. MicroRNAs as Biomarkers for PCOS Based on Follicular Fluid Content

Investigation of miRNAs in follicular fluid might reveal new targets for improving intrafollicular health and/or restoration of ovulatory dysfunction in PCOS. During oocyte retrieval, follicular fluid is easily available and could serve as an optimal, less-invasive source for miRNAs. Recent research has identified miRNAs in human [15,36] and mare follicular fluid [14]. Follicular fluid is in close proximity to the oocyte and consists of blood plasma components crossing the blood-follicular barrier, secretions from granulosa and theca cells and serves as an important microenvironment for the maturation, development and quality of the oocyte. Follicular fluid contains hormones, such as FSH, LH, GH, estrogens and androgens, growth factors, including TGF-β, inhibin, activin, anti-Müllerian hormone, as well as anti-apoptotic factors, such as Fas-ligands, and, lastly, proteins, peptides, amino acids and nucleotides [67].

Recently, two miRNA profiling studies using follicular fluid from PCOS women have been published, although with conflicting results [15,36]. Roth *et al.* performed a miRNA profiling study on PCOS patients fulfilling the Rotterdam criteria, undergoing IVF and compared them to healthy fertile oocyte donors. They found that 29 miRNAs were significantly differentially expressed between PCOS samples compared to controls. Out of the 29 miRNAs, hsa-miR-9, hsa-miR-18b, hsa-miR-32, hsa-miR-34c and hsa-miR-135a expression showed a significant increase in the PCOS samples compared to the controls. Subsequent cluster analysis classified the selected miRNAs into two distinct groups containing only PCOS patients in one of them, while the other group had all of the controls along with two PCOS samples, thus reflecting the heterogenic nature of PCOS [36]. In addition, based on *in silico* target site predictions and the association with PCOS phenotypes for the upregulated miRNAs, an mRNA expression profile of interleukin 8 (IL8), synaptotagmin I (SYT1) and insulin receptor substrate 2 (IRS2) in follicular fluid was further performed. All five miRNAs negatively correlated with the mRNA expression of IL8, SYT1 and IRS2 in the PCOS population, thus suggesting an inhibitory mode of action [36]. Sang *et al.* also isolated over 100 different miRNAs in follicular fluid from women with PCOS and healthy controls undergoing intracytoplasmic sperm injection (ICSI) treatment [15]. For the purpose of this review, not all of the identified miRNAs will be stated here, but a selection will be discussed. The PCOS patients were diagnosed based on the AES criteria. Construction of a small RNA library from pooled follicular fluid samples followed by sequencing revealed an abundant expression of mature miRNAs in follicular fluid. In accordance with a previous study [68], the eleven most highly-expressed miRNAs, hsa-miR-483-5p, -674-3p, -191, -193b, -320, -520c-3p, -24, -132, -146a, -222 and -1290, were present both free in solution and within micro-vesicles [15]. When comparing the miRNAs found in the follicular fluid of PCOS patients and healthy controls, only miR-132 and miR-320 showed a significantly lower expression level in the PCOS patients [15]. Specifically regarding miR-320, another study using follicular fluid and granulosa cells (GCs) from women with PCOS showed that the expression of miR-320 was higher in the PCOS group compared to controls, which contrasts the findings by Sang *et al.* [48]. Of interest, although miR-132 and miR-320 were observed to be decreased in the study by Sang *et al.*, this was not observed by Roth *et al.*, perhaps explained by the heterogenic nature of PCOS and the studied populations, differences in the control groups used or the general methodology. Thus, more studies are needed to resolve these divergent findings.

Interestingly, a recent genome-wide association study (GWAS) on the etiology of PCOS [69] identified one SNP in the intronic region of the high mobility group AT-hook 2 (*HMGA2*) gene and one SNP in the intergenic region near the Ras-related protein Rab-5B (*RAB5B*) gene. These genes are predicted targets of miR-132 and miR-320, respectively [15]. It remains to be elucidated if these genes in fact are true targets for these miRNAs and what their functions are in PCOS.

Noteworthy in the context of miRNAs serving as biomarkers, studies have shown that the follicular fluid content of different proteins and hormones, as well as follicular cell metabolism are altered in women of advanced maternal age [70]. Thus, one may speculate that the miRNA content, found in follicular fluid might also change in an age-dependent manner. MicroRNA profiling based on follicular fluid obtained from younger (median age 29.7, *n* = 3) and older (median age 40.3, *n* = 3) women undergoing IVF revealed that hsa-miR-21 was only present in young women’s follicular fluid, while hsa-miR-99b-3p, hsa-miR-134 and has-miR-190b were only found in, or expressed at higher levels, in the older women’s follicular fluid. Validation of the miRNA array profiling with more samples (*n* = 8 young *vs.* *n* = 6 old) by qRT-PCR followed the same pattern [13]. However, miRNAs found to be associated with reproductive aging in other studies were not altered in the study by Roth *et al.* Nevertheless, target gene predictions of the miRNA expression profiles found in follicular fluid in the studies by Roth and Sang suggest pathways involved in reproduction pathways, reproductive aging, carbohydrate metabolism, steroid synthesis, cellular growth, beta cell function, insulin signalling and cell communication [15,36].

In conclusion, comparing the miRNAs found in follicular fluid to the miRNAs found in the blood reveals the common occurrence of miR-186, miR-21, miR-155, miR-103, miR-19a and miR-16, although with different expressions levels and significance associated with PCOS. Moreover, the miRNA profile of follicular fluid varies between studies, highlighting that PCOS is a complex and heterogenic syndrome. Interestingly though, increased expression of miR-146 was found in serum from PCOS patients and also in follicular fluid by Roth *et al.* and Sang *et al.*, respectively. Adding to this, miR-222 and miR-24 were also found to be highly expressed in follicular fluid, as well as identified in PCOS serum. A differential expression, albeit in different directions, was observed for miR-320. Taken together, this still warrants further studies.

## 5. Possible Role for miRNA in the Abnormal Follicular Development and Function in PCOS

Many different theories have been brought forth in an attempt to explain the mechanisms responsible for the impaired ovulation, abnormal follicular development and excessive follicle formation commonly found in women with PCOS, but with varying results. An altered appearance and function of granulosa cells (GCs) with respect to FSH, LH and androgens has been proposed. Defects in steroidogenesis by the theca cells (TCs) and increased activation of primordial follicles, abnormal expression of anti-Müllerian hormone, increased follicle survival and/or a decreased apoptosis rate have also been reported. Many of the factors involved in these processes are still unknown and the mechanisms unestablished [71].

Total ovarian RNA from several species has been subjected to next-generation sequencing and cloning-based approaches. Thus, this has established that miRNAs are highly expressed in the ovaries of mice [21,23,25,28,29]. Furthermore, in mice, the degree of miRNA expression in the ovaries shows a genomic bias, with the X chromosome accounting for a high number of the miRNAs [72].

To investigate the miRNA profile in PCOS, different animal models have been used depending on the mechanisms being investigated. In a study by Hossain *et al.*, a rat model exhibiting both ovarian and metabolic characteristics of PCOS was used to study the miRNA expression. This PCOS model utilized exposure to 5α-dihydrotestosterone (DHT) to mimic the hyperandrogenic phenotype observed in human PCOS. Analysis of the ovaries from the DHT-induced rat PCOS model, using preformatted array plates, showed that 24% (equal to 79) out of 346 potential miRNAs were differentially expressed in the DHT-treated ovaries compared to untreated ovaries. Of the altered miRNAs found in the DHT-treated ovaries, 17 miRNAs were downregulated, whereas 72 miRNAs were upregulated compared with the control ovaries. A further characterization of the presence of miRNAs within the different ovarian compartments revealed that miR-222 was solely found in theca cells [24]. Thus, altered miRNA profiles seem to constitute part of the PCOS picture, even though complementary studies in human donor material needs to be added in order to tease out whether the alterations is a cause or consequence of PCOS.

More extensive studies of miRNAs in GCs have been performed compared to that of other ovarian cell types. Investigating the role of miRNAs for balancing apoptosis and proliferation, Sirotkin *et al.* found that apoptosis was induced in human GCs following transfection with 80 different miRNA constructs mimicking endogenous precursor miRNAs. Specifically, 11 of the miRNA constructs were associated with the accumulation of the apoptosis marker Bax and apoptosis induction [34]. The same study also investigated the proliferation of human GCs using proliferating cell nuclear antigen (PCNA) protein as a marker and the same miRNA constructs and found increased PCNA protein expression after transfection with eleven miRNAs constructs [34].

Apoptosis has also been induced in cultured human luteinized GCs following overexpression of pre-miR-23a. In this instance, anti-apoptotic X-linked inhibitor of apoptosis protein (XIAP) was reported to be downregulated, both at the transcriptional and translational level. XIAP prevents apoptosis by means of pro-apoptotic caspase inhibition; accordingly, the level of the cleaved form of caspase-3 was upregulated [73].

Studies on the apoptosis rate in mouse GCs, revealed that knockdown of miR-21 *in vivo* in equine chorionic gonadotropin (eCG)-treated mice resulted in an increased apoptosis rate. Furthermore, a decreased ovulation rate, as defined by the number of cumulus-oocyte complexes recovered from the oviduct, of the mice was shown [31].

Another miRNA of interest is miR-376, involved in primordial follicular assembly. Studies in fetal mouse ovaries show that miR-376a directly binds to the 3'UTR mRNA sequence of PCNA, thereby causing a reduction of PCNA on both mRNA and protein levels [74]. In this regard, an additional study in mouse shows that increased expression of miR-143 suppresses GC proliferation, thus inhibiting the formation of primordial follicles [75]. As miR-143 was also one of the differentially-expressed miRNAs found in follicular fluid from women with PCOS by Roth *et al.* [36], it constitutes a promising candidate for future investigations.

Further studies of GC proliferation in the mouse suggest miRNA involvement in the transforming growth factor-β (TGF-β)/Smad4 pathway, which is important for regulating cell growth, differentiation and development. In a study of isolated GCs from mouse pre-antral follicles treated with TGF-β1, it was found that the expression of miR-712, miR-224 and miR-764-3p was upregulated, while 13 miRNAs (including miR-143, miR-383 and miR-320) were downregulated. Specifically, the induction of GC proliferation through TGF-β1 was mainly mediated by the upregulated miR-224. Blocking the TGF-β receptor attenuated this effect, and Smad4 was identified as a target of miR-224. Thus, the increased TGF-β1-induced proliferation rate in GCs was mediated partly though the TGF-β/Smad pathway [47]. Of note, both Sing *et al.* and Roth *et al.* identified miR-224 in follicular fluid [15,36]. However, more studies need to be performed before concluding if the same pathway is affected in human folliculogenesis.

In addition, mmu-miR-224 has been identified as targeting pentraxin 3 (ptx3), an important protein involved in cumulus expansion [46,76]. Overexpressing mmu-miR-224 in the mouse cumulus oocyte complex indeed inhibited Ptx3 protein expression [46]. This miRNA could be of interest for further examination, as *PTX3* gene expression has been found highly upregulated during ovulation in GCs isolated from women following controlled ovarian stimulation. Perhaps, PTX3 levels could serve as a marker for final oocyte maturation [77]. Further, with regards to PCOS, *PTX3* mRNA expression in PCOS patients correlates with fertilization processes [78] and could possibly be used as a biomarker for oocyte quality. However, consensus has not yet been reached; a study report that plasma PTX3 levels in women with PCOS is reduced [79], whereas yet another study shows PTX3 levels to be elevated in PCOS and positively correlated with insulin resistance [80]. As such, more research needs to be done before reaching a conclusion on the role of miR-224 in PCOS and whether altered PTX3 levels are a cause or consequence of the disease.

Stages of early and progressive atresia in follicles have been assessed in pigs using a microarray approach [33]. A decreased rate of follicular atresia has been observed in cultured human ovarian tissue from PCOS follicles [81]. It was found that miR-26b, an miRNA also found in human follicular fluid [15], was upregulated during follicular atresia. When further investigating possible functions of miR-26b through culturing the porcine GCs and transfecting with miR-26b mimics, it was shown that the apoptosis rate increased. It followed that miR-26b targeted and repressed ataxia telangiectasia mutation (ATM) at the mRNA level, a protein known to coordinate DNA repair. TUNEL assays performed after miR-26b transfection showed an increase in DNA breaks, thus explaining the increase in apoptosis [33].

Taken together, altered expression of ovarian miRNAs might play a role in the processes determining the fate of granulosa cells (proliferation and differentiation *vs.* apoptosis), and this might lead to the hyper-proliferating granulosa cells, as seen in PCOS. The roles and mechanisms of miR-224, miR-320 and miR-383 in GCs during folliculogenesis in general and in PCOS remain unknown.

## 6. Infertility, an Important Feature of PCOS and the Involvement of microRNAs

The importance of microRNAs in regards to fertility, follicle development and oocyte maturation has become evident using the Dicer1 knock-out mouse. Dicer1 is an, RNase III enzyme involved in the processing of pre-miRNA into the shorter miRNA duplex [82] and, thus, important for microRNA biogenesis. Knock-out of Dicer results in lethality early in development, while conditional ovarian tissue-specific knock-out models of Dicer result in infertile female mice, low ovarian weight, low ovulation rate, abnormal estrous cycles and dysregulation of development-related genes. Furthermore, defects in meiotic progression due to defective spindle formation, defects in the development and function of the ovarian corpus luteum, due to abnormal angiogenesis in the corpus luteum, and the formation of fluid-filled cysts on the oviducts are also evident in the conditional knock-out model. Studies describing Dicer knock-outs have recently and extensively been reviewed elsewhere [83,84,85] and will not be described further.

Isolated blastocysts from women referred to IVF, either due to polycystic ovaries or male factor infertility, revealed that the expression of six miRNAs (hsa-let-7a, hsa-miR-19a, hsa-miR-19b, hsa-miR-24, hsa-miR-92 and hsa-miR-93) was significantly decreased in the blastocysts from the PCOS patients compared to blastocysts from healthy fertile donor oocyte controls [40]. Heatmap analysis for the expression level of the six miRNAs showed that the heatmap profile of women with polycystic ovaries was different from that of the fertile donors and women from couples having male factor infertility; the latter shared a close resemblance to each other. The morphology of the blastocysts was similar for the three groups. Further, three predicted target genes were chosen for mRNA expression. The expression of the miR-19a target gene ARIH2, important for cell differentiation, showed an upregulation only in the PCOS group, with a simultaneous decrease in miR-19a. Likewise, the KHSRP and NFAT5 genes, encoding an mRNA decay promoting factor and a transcription factor, respectively, and both targets for miR-24, showed an upregulation in both of the infertile groups, together with an observed decrease of miR-24 [40]. With the exception of hsa-let-7a, all of the miRNAs were also detected in human follicular fluid, but the expression levels displayed a great variation, never reaching a significant difference between PCOS women and healthy controls.

Maternal age-related infertility and miRNA expression levels were investigated using human blastocysts derived from younger (mean 26.4 years) and older (range 40–44 years) oocyte donors [86]. Out of 42 differentially-expressed miRNAs, the majority were increased in older women. Eleven miRNAs (miR-15b, miR-18a, miR-184, miR-195, miR-20b, miR-212, miR-222, miR-367, miR-515-5p, miR-518a-3p and miR-93) were only present in the older study population, and miR-93 expression was particularly increased in blastocysts showing abnormal chromosomes [86]. Maternal aging is a risk factor associated with human infertility, which is associated with an altered miRNA profile.

## 7. MicroRNAs and Ovarian Hormone Synthesis

The ovarian cycle is controlled by hormones released from the hypothalamic-pituitary-ovarian axis, and an altered secretion has been observed in women with PCOS. Although androgen excess is a prominent feature of PCOS, androgens have proven important for female reproduction, since a lack of androgen receptors and, hence, the inability of androgens to stimulate their targets, result in reproductive defects [87,88,89]. The level of free, and, thereby, bioavailable and active, testosterone is only around 1%–2%, whereas the rest is either bound to sex-hormone binding globulin (SHBG) or albumin. Women with PCOS often display low levels of SHBG [90], and in addition to this, hyperinsulinemia can also decrease the level of SHBG [91], thus increasing the levels of bioactive testosterone. Within the ovaries, theca cells synthesize androgens, when stimulated by luteinizing hormone (LH). Ovarian GCs, under the influence of follicle-stimulating hormone (FSH), produce the enzyme, aromatase, which is able to convert testosterone and androstenedione to estrogen. Within peripheral adipose tissue, aromatase also converts testosterone to estrone.

The influence of specific miRNAs on the release of steroid hormone from ovarian cells has been demonstrated by several cell studies across a variety of species.

Cultured primary human ovarian GCs transfected with 80 different miRNA overexpression constructs revealed that 36 miRNAs correlated with inhibited progesterone release, while 10 miRNAs increased the release of progesterone after transfection. Moreover, 57 of the constructs inhibited the release of testosterone and 51 miRNA constructs correlated with reduced estradiol secretion. Only miR-107 was found to increase the release of testosterone [35].

Transfection with miRNA-24 mimics in the steroidogenic human granulosa-like tumor cell line, KGN, resulted in decreased estradiol secretion, while transfections with miR-132, miR-320, miR-520c-3p and miR-222 mimics increased estradiol secretion. Moreover, miR-24, miR-193b and miR-483-5p transfections also correlated with decreased secretion of progesterone [15].

Studying ovulation and luteinization, miRNA expression profiles were investigated in mice that had been stimulated with eCG to induce follicular development. Ovulation was then induced using human chorionic gonadotropin (hCG) injection after 46 h of eCG-stimulation. The following microarray analysis was based on isolated mouse mural GCs collected previous to and 4 h post hCG-stimulation. The microarray analysis revealed that 13 miRNAs were differently expressed between the two different time points, with miR-21, miR-132 and miR-212 being specifically upregulated by hCG. It is known that hCG and LH upon binding to the LH receptor activate the cAMP signal transduction pathway. In order to elucidate the role of miR-132 and miR-212 in ovulation and luteinization, mural GCs were exposed to cAMP *in vitro*. Confirming *in vivo* results, cAMP induced the expression of the two miRNAs *in vitro*. However, knockdown of miR-132 and miR-212 did not affect cAMP-induced steroidogenesis *in vitro* [30].

### 7.1. MicroRNAs Targeting Steroid Receptors

Increased expression of androgen receptors (AR) has been identified in women with PCOS [92], and even though androgen excess is an important feature in the pathogenesis of PCOS, androgens and their receptors have proven important for normal ovarian function, as observed in the AR-knockout model, having defective folliculogenesis. AR is expressed predominantly in GCs of growing ovarian follicles [93], but can also be found in other reproductive cells, as well as in non-reproductive tissues.

The luteinizing hormone/chorionic gonadotropin receptor (LHCGR), as well as miR513a-3p are both expressed in human ovarian GCs. LHCGR is a predicted target of miR-513a-3p, and downregulation of LHCGR expression by miR-513a-3p has been observed [94]. A next generation sequencing approach in human GCs identified three novel and previously un-annotated miRNAs. The three miRNAs; miRNA-7973-1, miRNA-7973-2 and miR-548ba, were predicted to originate from the FSHR and CYP19A1 genes, respectively. Furthermore, one of these miRNAs was predicted to target the AVBR2B and SMAD2 genes, which are involved in FSHR mRNA expression in GCs [95]. Isolated GCs from oocyte retrieval in women undergoing IVF have been shown to express miR-125b. Using a GC-specific androgen receptor (AR) KO mouse model, the expression of the anti-apoptotic miR-125b was enhanced by extra-nuclear AR signaling. The increase was found likely to contribute to androgen-induced follicular survival by a decrease in follicular atresia. Secondly, the protein levels of FSHR, but not mRNA, were found to be increased by androgens, potentially contributing to follicles being more sensitive to FSH, hence stimulating androgen-mediated follicle growth [96].

miR-136-3p expression was increased in a study of rat ovaries and isolated GCs following an ovulatory dose of LH-human chorionic gonadotropin (hCG). Simultaneously, mRNA levels of the hCG receptor (LHR) were reduced, and thus, miR-136-3p was suggested to target LHR [97]. Another study in rat following hCG stimulation revealed an increase in ovarian miR-122 with a concomitant decrease in LHR mediated via the cAMP/PKA pathways. In this instance, though, a simultaneous increase in LHR mRNA binding protein (LRBP) was also found, at both the mRNA and protein level. This observation was also confirmed using a similar approach with human GCs [98].

Estrogen receptor alpha knockdown adult female mice display cystic ovaries, high levels of LH and ovulation abnormalities. The estrogen receptor 1 (ESR1) gene is a predicted target for miR-222, miR-193b and miR-520c-3p [99]. At least in studies of breast cancer, miRNA-222 has been shown to negatively regulate the estrogen receptor alpha at the protein level [45], while miR-193b regulates estrogen signaling [100]. Interestingly, miR-222, -520c-3p and 193b can influence not only the estrogen receptor, but also steroid secretion, making these miRNAs very interesting candidates for further studies. It is possible that an abnormal expression of estrogen receptors or aberrant estrogen receptor signaling might contribute to the pathogenesis, the abnormal follicular development and the infertility seen in PCOS [101].

### 7.2. MicroRNAs Targeting Steroid Synthesis Enzymes

The biosynthesis of steroid hormones relies on different genes, including StAR, CYP19, CYP11A and CYP17. Thus, miRNAs affecting these genes might play a role in the perturbed homeostasis of steroid hormones commonly seen in PCOS. In the search for PCOS candidate susceptibility genes, results regarding CYP11A are presently conflicting. One large patient-based study find no associations linking CYP11A to the disease [102] while others report a relationship between CYP11A promoter variants and raised testosterone levels [103,104,105].

The production of estrogens relies, in part, on the enzyme, aromatase. Aromatase is encoded by the CYP19A1 gene, with tissue-restricted expression, e.g., within the ovaries, where the enzyme is ultimately produced in GCs under the influence of FSH [106]. To our knowledge, studies investigating potential miRNAs regulating CYP19A1 expression in GCs have only been performed in pigs and mice.

When GCs isolated from porcine ovaries were treated to overexpress miR-378 at high levels, a subsequent decrease was observed of aromatase protein following and, hence, reducing estradiol concentrations in the media of the cultured GCs [107]. Another study in mouse GCs reveals that miR-181a also targets CYP19A1, thereby downregulating estrogen biosynthesis [108].

On the other hand, several miRNAs have also been shown to associate with increased estradiol levels. Following overexpression of miR-133b in mice GCs stimulated with FSH, estradiol levels were increased with a simultaneous increase of both CYP19A1 and StAR mRNA levels [109]. A predicted target of miR-133b was the forkhead box L2 (Foxl2), which functions as a transcriptional repressor of CYP19A1 and StAR. Furthermore, the *FOXL2* gene has been shown to be vital for female reproduction [110,111]. In accordance, loss-of-function studies using miR-133b inhibitors decreased the estradiol concentration in a KGN cell line, as well as in the mouse GCs. Thereby, miR-133b may play a role in FSH-induced estrogen secretion by indirectly increasing the expression of the steroidogenesis-associated genes StAR and CYP19A via repression of Foxl2 [109]. Increased release of estradiol from mouse GCs via an enhanced expression of CYP19A1 has also been reported following upregulated levels of miR-383 in a dose-dependent manner. The mechanism of action for miR-383 was predicted to be mediated through an inhibition of the transcription factor RBMS1 gene and its downstream target c-Myc, hence influencing estradiol release in GCs. No effect on progesterone production, GC proliferation or apoptosis was reported [50]. As upregulated miR-383 levels have previously been detected in women with PCOS 34, this miRNA could be interesting to study further, as, perhaps, it may be a mediator of the pathologically-elevated levels of estrogen commonly detected in women carrying the disease. A third study shows that the level of CYP19A1 mRNA increased in mouse GCs treated with either miR-224 or TGF-β1, without any effect on the CYP11A1 mRNA level. Overexpression of miR-224 resulted in increased estrogen release, while knockdown of miR-224 had the opposite effect, indicating that miR-224 could play a part in steroid production. One predicted target gene of miR-224 is Smad4, and miR-224 was able to negatively regulate Smad4 (a mediator of TGF β signaling), thus regulating GC proliferation and estradiol release [47]. Interestingly, studies have revealed that miR-224 transcription in mouse GCs is regulated by the two transcription factors Tp53 and the p65 subunit of NFκβ, through transcriptional inactivation of the GABRE (miR-224 host gene) promoter. Smad4 is targeted by miR-224, and knockdown of Tp53 and NFκβ p65 led to lower Smad4 protein levels, while GC proliferation and estradiol production increased [112].

Understanding how miRNAs contribute to androgen production has great potential with respect to steroid-related disorders, such as PCOS.

## 8. MicroRNAs, Insulin Sensitivity and Insulin Resistance

Most women with PCOS suffer from some degree of insulin resistance and hyperinsulinemia. Even though insulin resistance is not a diagnostic feature of PCOS, as many as 38%–88% of PCOS women are either found to be obese or overweight. There is a disadvantageous interaction between obesity, insulin resistance and PCOS, with obese women with PCOS being more insulin resistant. Studies in regard to lean women with PCOS and the presence of insulin resistance have proven inconsistent (reviewed by [113]).

In a study of primary adipocytes from women with PCOS, it was found that the level of GLUT4, the major insulin-dependent glucose transporter, was positively correlated with insulin sensitivity. Further, miR-93 was predicted to target GLUT4, and their respective expression levels were inversely correlated. This correlation was further supported by overexpression and knockdown studies of miR-93. However, although miR-93 might play a role in determining insulin sensitivity via GLUT4, miR-93 itself was associated with PCOS status [44].

Hyperinsulinemia could potentially contribute to the hyperandrogenism found in women with PCOS. Insulin has been shown to activate the enzyme CYP17 and work synergistically with LH within porcine theca cells to increase cAMP concentration, thus leading to an activation of StAR and the production of androgens [114]. Moreover, insulin has also been observed to induce follicular arrest [115,116]; however, the mechanisms behind follicular arrest are not fully understood. An improvement of insulin sensitivity may result in a more controlled glucose metabolism, reduced androgen levels, better ovulation rates and, hence, improved fertility.

Since their discovery, several miRNAs have been identified and found to be differentially regulated in a variety of animal models of diabetes. In a hyperglycaemic, T2D rat model, miR-222 and miR-27a were shown to be upregulated in adipose tissue [117]. Additionally, miR-29 paralogs have also been found upregulated in adipose tissue in a diabetic mouse model and associated with increased insulin resistance in the mouse-derived 3T3-L1 adipocyte-like cell line [118]. In another study, 3T3-L1 adipocytes were rendered insulin resistant by treatment with high levels of glucose and insulin. It followed that the expression levels of miR-320 displayed a 50-fold increase. The insulin sensitivity was then restored upon the addition of anti-miR-320 oligos, with a resulting upregulation of GLUT4 expression and increased insulin-mediated glucose uptake [49]. If the pathway for miR-320 is similar in humans, it could potentially serve as a target to improve insulin sensitivity, as miR-320 has been found to be very abundant in follicle fluid from women with PCOS [15].

To improve insulin sensitivity in PCOS women, insulin sensitizing drugs, such as metformin, are prescribed. Metformin decreases hepatic glucose production while stimulating glucose uptake in insulin-sensitive tissues and improves lipid metabolism. Taken together, the medication decreases insulin resistance with several positive effects on androgen levels, ovulation and, subsequently, fertility [119]. The expression pattern of certain miRNAs might also be regulated by the administration of metformin. The expression level of miR-221 and miR-222 was found to be elevated in the internal mammary arteries (IMAs) of subjects with T2D, while patients on metformin displayed an expression level similar to non-diabetic patients [120]. Others have studied the effects of metformin on altered miRNA expression in various cancers [121,122].

Metformin is capable of changing global miRNA expression patterns, as shown in the aforementioned studies in metformin-treated cancer samples. Furthermore, the finding that metformin is able to induce Dicer expression [123] might establish a link between the metabolic action of the drug and altered miRNA expression.

## 9. Conclusions and Further Perspectives

In summary, the pathophysiology of PCOS is not easily summarized, since it involves areas within gynaecology, endocrinology and diabetology. Depending on which PCOS criteria are used, different phenotypes arise and, in addition, the varying networks of microRNAs contribute to the complexity. The notion that one miRNA may have several mRNA targets and that one mRNA 3'UTR may be regulated by multiple miRNAs complicates matters further. As such, our current knowledge of how miRNA might fine-tune the events leading to increased ovarian cell proliferation and/or apoptosis, abnormal folliculogenesis, altered steroidogenesis or adipocyte dysfunction is not yet complete, and more functional miRNA-mRNA studies are needed. With the current knowledge, it is not possible to distinguish whether an altered miR expression profile is the cause or a consequence of PCOS. However, it appears that microRNAs could be used as biomarkers distinguishing PCOS patients from normal menstruating women or to identify sub-phenotypes within this heterogeneous syndrome. Furthermore, insight into the interplay between epigenetic regulation and miRNAs leading to an altered miRNA expression could perhaps help to explain the pathogenesis behind the disease. The dynamic expression of miRNAs has been shown to be influenced by several factors, such as LH/hCG, FSH, androgens and cAMP *in vitro.* Caution needs to be exerted when interpreting *in vitro* and *in vivo* findings, since miRNAs might be regulated differently by the two systems. Although miRNAs have been identified in the ovary and different ovarian compartments, as well as in the circulation, little is known about the roles of miRNA in PCOS pathology. Of interest, PCOS subjects are often undergoing IVF treatment and/or receiving metformin, which might affect the miRNA expression profile. Currently, only a limited number of studies have aimed to extensively profile miRNA expression and function within a PCOS study population, and the results are at times, contradictory. However, it follows that a resilient motivation should arise to further research the actions and regulations of miRNAs to clarify their role in PCOS.
